# Depression among Older Adults in Indonesia: Prevalence, Role of Chronic Conditions and Other Associated Factors

**DOI:** 10.2174/17450179-v18-e2207010

**Published:** 2022-09-05

**Authors:** Yvonne Suzy Handajani, Elisabeth Schröder-Butterfill, Eef Hogervorst, Yuda Turana, Antoninus Hengky

**Affiliations:** 1 School of Medicine and Health Science, Atma Jaya Catholic University of Indonesia, Jakarta, Indonesia; 2 University of Southampton, Southampton, United Kingdom; 3 School of Sport, Exercise & Health Sciences, Loughborough University, Loughborough, United Kingdom; 4 Center of Health Research, Atma Jaya Catholic University of Indonesia, Jakarta, Indonesia

**Keywords:** Prevalence, Depression, Older adults, Associated factors, Chronic condition, Health condition

## Abstract

**Background:**

Depression is one of the most common illnesses worldwide, with a prevalence of 5.7% among older adults aged over 60. Depression is a severe health condition that can significantly affect the quality of life.

**Objective:**

The objective of this study is to investigate the determinant factors of depression among older adults in Indonesia.

**Methods:**

Data of 4236 adults of 60 years old and over were taken from the fifth wave of the Indonesian Family Life Survey (IFLS-5). Sociodemographic and multiple health-related variables collected through interviews and measurements were analyzed. Multivariate logistic regression was used to evaluate depression and its associated factors.

**Results:**

The prevalence of depression assessed using ten questions from the Center for Epidemiologic Studies Depression Scale (CES-D 10) was 16.3%. Significant associated factors for depression were moderate and low subjective economic status, living in Java or other regions outside Sumatra and Java, no life satisfaction, self-perceived as having poor health, having dependency (IADL scores), and experienced falls and insomnia. Among chronic conditions, stroke, arthritis, and hearing impairment were also more common in depressed older adults.

**Conclusion:**

Predictors of depression identified in this study may be used to help prevent and improve depression in Indonesian older adults, especially those who live on Java. Improvement in healthcare, especially in the prevention and rehabilitation of stroke, arthritis, possible frailty (falls and dependency), hearing impairment, and insomnia, concurrent with early detection of depression in these chronic conditions, may help create a better quality of life among Indonesian older adults.

## 1 INTRODUCTION

Depression is one of the most common illnesses worldwide, with a prevalence of 3.8% among the general population and 5.7% among older adults aged 60 and over [1]. Depression is a severe health condition, especially if recurrent and of moderate to extreme intensity, which can significantly affect the person's quality of life [2]. Depression becomes a burden for the person, family, and society due to lowered productivity and higher healthcare costs [3, 4]. The prevalence of depression in older adults may be underestimated because it tends to be unrecognized and untreated compared to younger individuals [2]. In Indonesia, the number of older adults has increased over the past decades, with an estimated 16 million older adults (6.1% of the population) in 2019 [5]. This sizeable aging population faces various challenges, including disability, multiple chronic diseases, frailty, and especially depression [2, 6, 7]. According to Indonesia's basic health research, the prevalence of mental health disorders in Indonesia has increased from 6% in 2013 to 9.8% in 2018. In the same study, depression among persons older than 15 years old in 2018 was estimated at 6.1%, with only 9% receiving treatment for the condition in Indonesia [8].

Depression prevalence was lower in individuals with higher socioeconomic status, better self-reported health, more social capital, and functional independence in multiple studies [9-14]. In addition, engaging in physical activity was found to act as a protective factor against depression [15-17]. Various studies have shown that depression increases with multimorbidity, frailty, and dementia. Chronic diseases, including hypertension, diabetes, stroke, heart disease, kidney disease, and liver disease, confer a risk of depression over time [9, 10, 18]. People with visual and auditory impairment also have a higher prevalence of depression than the average population [19-21].

There is limited research on depression and its associated factors among the older adult population in Indonesia, and some studies only involve small samples [22-24]. Due to increasing trends and the high disease burden, more studies regarding depression in Indonesia have become necessary. The Indonesia Family Life Survey provides large representative data from various regions in Indonesia; data analysis on older people from these surveys might help create better-targeted strategies to prevent and improve depression among older Indonesian adults. This study aims to investigate factors associated with depression among older adults in Indonesia.

## 2 MATERIALS AND METHODS

### 2.1 Study Design and Participants

Indonesia Family Life Survey (IFLS) is the only large-scale longitudinal survey available for Indonesia. The fifth wave of the IFLS was collected cross-sectionally from the end of 2014 to early 2015 and involved data related to individuals, families, households, and communities. The sampling method was a multistage stratified sampling design. The total number of participants included in the analysis was 4236 older adults aged 60 years and older residing in various regions of Indonesia [25]. IFLS-5 involved participants from previous IFLS (IFLS 1 to 4) with recontact rate of 90.5%. These high re-interview rates were obtained in part because of committed tracking and interviewing of individuals who had moved or split off from the origin IFLS-1 households [26]. IFLS involved participants stratified in provinces and urban/rural locations. Provinces were determined to maximize representation of the population, capture Indonesia's cultural and socioeconomic variations, and still be cost-effective. Therefore, this study included 13 out of 34 provinces comprising 83% of the population, which are four provinces on Sumatera (North Sumatera, West Sumatera, South Sumatra, and Lampung), all five of the Java provinces (Jakarta, West Java, Central Java, Daerah Istimewa Yogyakarta, and East Java), and four provinces covering the remaining major island groups (Bali, West Nusa Tenggara, South Kalimantan, and South Sulawesi). Within each of the 13 provinces, enumeration areas (EAs) were randomly chosen from a nationally representative sample frame used in the National Census. Within each EAs, households were randomly selected based upon National Census listings obtained from the regional national statistics center office. The household survey questionnaire was divided into several books depending on the participants’ age and research variables. We used books 3A and 3B questionnaire data, which contain individual-level sociodemographic and health data from adult respondents, and book US, which contains physical health assessments of the individuals. In IFLS-5, the obtained data were input electronically in the field after the interview by the trained interviewer to a computer-assisted personal interview system (CSPro). All of the available data of the participants were included.

### 2.2 Measures

Multiple validated and standardized sociodemographic and health variables were assessed and are defined in (Table **1**) [27-33]. These variables were considered to be included because they involved variables usually associated with other health problems, particularly in older adults. Sociodemographic factors included age, sex, marital status, education level, residential status, region, and subjective economic status. Assessment of variables mostly used validated measurements to avoid bias. Body mass index (BMI) was calculated using measured height and body weight taken using standard instruments and classified using Asia Pacific Classification [30]. Depression was assessed using the Center for Epidemiological Studies Depression Scale (CES-D) [27]. Depression has a huge societal impact, making accurate measurement paramount. While several available measures exist, the Center for Epidemiological Studies Depression Scale (CESD) is a popular assessment tool with wide applicability in the general population. CES-D was shown to have high validity and reliability from exploratory factor analysis and confirmatory factor analyses with internal consistency reliability (Cronbach’s alpha = 0.84) [34]. Life satisfaction, subjective health status, social capital, tobacco use, chronic conditions, and history of falls were obtained based on specific questions in the questionnaire. The functional ability was assessed using Katz’s Activity of Daily Living (ADL) and Lawton's Instrumental Activity of Daily Living (IADL) [28, 29]. The physical activity was assessed using International Physical Activity Questionnaire (IPAQ) short version (IPAQ-S7S) [31]. The cognitive function was evaluated using the Telephone Survey of Cognitive Status (TICS) [32]. Insomnia was assessed using Patient-reported Outcomes Measurement Information System (PROMIS) sleep disturbance and sleep impairment measure [33].

### 2.3 Statistical Analysis

Comparisons of characteristics between groups of dependent variables were analyzed using bivariate analyses (binary logistic regression) to assess the association between the dependent and each independent variable. Variables with less than 0.2 p-values were fitted to the multivariable logistic regression. Odds ratios with 95% CI were calculated, and statistical significance was considered at p-value < 0.05 in the multivariable logistic regression to control for any confounding. The IBM SPSS software version 22 (IBM, New York, USA) was used to perform the analyses.

## 3 RESULTS

### 3.1 Characteristics of Older Adults

A total of 4236 older adults were involved in the analysis, and no missing data was found. The prevalence of depression assessed using ten questions from the Center for Epidemiologic Studies Depression Scale (CES-D 10) was found to be 16.3% in Indonesia (Table **2**). The majority of the respondents (70.3%) were aged between 60 to 69, more than half (50.3%) were female, two-thirds (66.6%) were married or cohabiting, 85% had low education (<9 years), 40% reported a moderate subjective economic status and more than half (50.8%) lived in urban areas, mostly (78.2%) on Java conform census. The majority (82.2%) of the respondents reported satisfaction with life and self-perceived themselves as healthy (65.4%), most were active in social activities (82.3%), about a third of the respondents were smokers (33.3%), and many engaged in low physical activity (47.2%). From the self-reported history of medical illness, most respondents stated they had good cognitive function (86.0%), and less than 5% of respondents had experienced a stroke, heart disease, hearing impairment, or cancer. More than 10% of the respondents had arthritis, visual impairment, and hypertension, which was the most prevalent (27.6%). Almost half (46.6%) of the respondents experienced one or more chronic diseases. Furthermore, 10.5% of respondents had insomnia, 12.7% reported dependency in activities of daily life (ADL), and a quarter (25.6%) had dependency in instrumental activities of daily living (IADL), while 12.0% had experienced falls.

### 3.2 Factors Associated with Depression

The data showed that depression was significantly associated with having moderate or low subjective economic status, living in Java and other regions, no life satisfaction, being self-perceived as unhealthy, having had a stroke, reporting arthritis, hearing impairment and insomnia, dependency (IADL) and falls. However, depression had no significant association with age, sex, marital status, education, residential status, social capital, tobacco use/smoking, physical activity, BMI, cognitive function, heart disease, hypercholesterolemia, diabetes, hypertension, visual impairment, cancer, chronic conditions and dependency (as assessed by ADL).

Older adults with moderate and low subjective economic status were 1.34 times and 1.64 times more likely to have depression than older adults with high subjective economic status (AOR = 1.34, 95% CI: 1.05-1.76) and (AOR =1.64, 95% CI: 1.28-2.11). Respondents from Java and other regions outside Java and Sumatera were 1.80 times (95% CI: 1.33-2.44) and 2.25 times (95% CI: 1.52-3.31) more likely to have depression than respondents from Sumatera. People who were dissatisfied in life and self-perceived as unhealthy were 1.75 times (95% CI:1.42-2.17) and 1.85 times (95% CI:1.53-2.24) more likely to have depression. Moreover, older adults with a history of medical illness, such as stroke, arthritis, and hearing impairment, were 2.45 times (95% CI: 1.60 -3.74), 1.59 times (95% CI: 1.27-2.00), and 1.69 times (95% CI: 1.03-2.05) more likely to be depressed than those who did not experience this disorders. Furthermore, respondents who experienced insomnia, dependency (IADL), and falls tended to have depression 5.3 times (95% CI: 4.24- 6.62), 1.68 times (95% CI: 1.37-2.05), and 1.57 times (95% CI: 1.23-2.01) more than those who did not experience these issues (Table **3**).

## 4 DISCUSSION

The prevalence of depression in our study was high (16.3%). This number was higher than that reported in the general population from the 2018 Indonesia Basic Health research (6,1%) [8]. Studies of older adults from a Portuguese national cohort (11.8%), the South Africa nation-wide survey (4%), and a meta-analysis by Lim *et al*. (12.9%), all showed lower prevalence. In comparison, studies from a nationwide survey in South Korea (27.8%) and a rural area in Thailand (18.5%) showed higher prevalence [9, 10, 35-37]. Different assessment methods of depression may cause variations between these studies, and alternatively, different cultures and sampling methods could be responsible for discrepancies. Both studies in South Korea and Thailand used Geriatric Depression Scale (GDS), but a different cut-off was established, with the South Korean study using ≥8 as a cut-off while the Thailand study using ≥13. This may explain the higher prevalence of depression in the South Korean study. The South African study used the World Mental Health Survey version of the Composite International Diagnostic Interview, and Indonesia basic health research used the Mini International Neuropsychiatry Interview; both showed lower prevalence than other studies. A meta-analysis by Lim *et al*. also showed that self-reported assessments tend to have a higher score than clinical diagnostic interviews. Diagnostic interviews adopted stricter criteria to differentiate individuals with clinical depression from those with only depressive symptoms.

Among demographic factors, there was an association with living in Java and other regions, while being older was not associated with depression. Many studies have shown that older age confers a higher risk, but this was not found in a meta-analysis by Maier *et al*. [18]. The insignificant result between older age and depression may be explained by the fact that older age is related to declining physical health and disability, which results in depression, and is not age-independent [10]. However, there was no sex difference in depression in our study. However, many studies have shown that older women tend to have depression more commonly than men [18, 36]. Given bio-psychosocial aspects, women have a higher concentration of serotonin that plays a role in anxiety and stress; they also tend to have a dysregulated hypothalamic-pituitary-adrenal axis in response to stress compared to men; women are also more likely to have experienced sexual and emotional abuse, and in the Asian and many other cultures, women’s role is restricted to home duty and child care, which are associated with depression. Women were also reported to be more likely poorer or have less control over economic resources in the household [5].

Depression was also more prevalent in participants with functional dependency as measured by IADL, having moderate and low socioeconomic status, low life satisfaction, and being self-perceived as unhealthy. These findings are consistent with many studies, including a meta-analysis [9, 16, 38, 39]. Having a functional disability could increase the prevalence of having clinical depression and worsen the existing depression [40]. The declining physical function creates uncertainty and hopelessness by being dependent in older adults [41, 42]. Being poor by the subjective judgment of the participant and/or from income/wealth aspects (objective poverty) predisposes older adults to depression. Having a low income may lead to poor living conditions and multiple serious life events, which increase the higher prevalence of depression. Men tend to be more affected by low socioeconomic status than women because, traditionally, men are the head of the family and responsible for family needs [42]. On the other hand, women usually outlive men and then live in poorer households, so they are more likely to experience poverty in later life [43]. However, as stated, we found no gender differences in depression. In a study done by Sasaki *et al*., subjective economic status had a stronger association than objective economic status. Some reasons for this phenomenon were health disparities and negative psychological consequences (stress-related dysregulation of the hypothalamic-pituitary-adrenal axis) due to differences in subjective economic status. Subjective economic status might represent a cognitive estimation of current economic status and extend it to the past and future prospects [44]. Having dissatisfaction in life and self-perception as being unhealthy probably have a bidirectional relationship with depression.

Having a low physical activity or sedentary life has a bidirectional relationship with depression. Depressive individuals tend to be less physically active, even formerly active individuals [17]. Having an active lifestyle has been associated with fewer depressive symptoms in other studies. The relation between depression and physical activity may be mediated by several variables, such as social relations and engagement, chronic conditions, and functional ability [45]. Older adults who have a disability may also be unable to do certain activities. Despite these findings, our study did not show a significant association between physical activity and depression. A meta-analysis by Huang *et al*. showed that older adults with dementia had an increased probability of depression compared to those with good cognitive function [46]. However, a systematic review by Maier *et al*. concludes that it is difficult to extrapolate these associations due to heterogeneous results between the studies [18]. In a study by Carta *et al*., performing moderate exercise improves cognitive function in healthy older adults. Due to bidirectional relationships between physical activity and cognitive function with depression, improvement in either variable might improve depression in older adults [47].

Insomnia was associated with depression in our study. A longitudinal study by Li *et al*. also confirmed our findings [48]. Two prospective cohort studies by Lee *et al*. and Baglioni *et al*. found that insomnia significantly increased depression in nondepressed older adults at baseline in the subsequent year [49, 50]. In the study by Lee *et al*., persistent insomnia increased depression risk rather than intermittent insomnia. A meta-analysis by Maier *et al*. also showed that insomnia, especially early insomnia, was significantly associated with depression [18]. Our assessment of insomnia has a limitation in that we could not differentiate insomnia into multiple types for further analysis. Insomnia and depression have a complex relationship. Depression diagnoses usually have criteria for sleep disturbances, while insomnia could cause alterations in emotional response, resulting in depression. Other studies also show that insomnia endorses maladaptive sleep beliefs that increase the individual’s sense of hopelessness, which also results in depression [51].

Our findings regarding stroke showed an increased prevalence of depression significantly, which has also been demonstrated by other studies [52-55]. The prevalence of post-stroke depression was around 31.1%, with more women affected by it [54]. The pathogenesis of poststroke depression itself is multifactorial, involving complex biological, social, and psychological mechanisms [56]. The higher number of depression in stroke patients may also be mediated by long-term hospitalization, isolation, and problems adapting to new conditions, such as significant disability and communication difficulties [55]. A longitudinal study by Thomas *et al*. showed that two-thirds of poststroke patients remained depressed at follow-up between 7 to 13 months and became even more depressed [53]. Lower perceived control of recovery and difficulty in communication contributed to this phenomenon. One of the problems is that depressive symptoms usually also involve somatic complaints, which can also be affected by the presence of stroke, so it is difficult to determine an accurate diagnosis [55].

In our study, more older people with arthritis suffer from depression than those without arthritis. According to Mortality and Morbidity Weekly Report (MMWR), in 2018, around 22.7% of US adults were diagnosed with arthritis, and 22.5% and 12.1% were suffering from anxiety and depression [57]. Based on age-specific prevalence, anxiety and depressive symptoms were more common in those between 18-44 years than in people over 65 years in our study. Visual and auditory impairment was significantly associated with depression in multiple studies, but in our study, only auditory impairment was associated with depression [19, 58, 59]. A study by Chou *et al*. found that visual impairment has a more robust impact than hearing impairment because it tends to be more obvious; it is also perceived as worse than hearing loss and wider restrictions [19]. A national cohort study by Kim *et al*. showed that mild to moderate hearing loss has little impact on depression, but in our study, we did not classify the hearing impairment because it was self-reported [60]. In treating patients with visual or hearing impairment, the healthcare providers often do not address the mental health aspects, including depression.

## CONCLUSION

The prevalence of depression among older adults in Indonesia is still very high. Many factors contribute to depression, such as living in Java and other regions, being dependent, having moderate to low subjective economic status, having no life satisfaction, being self-perceived as unhealthy, and having experienced stroke, arthritis, hearing impairment, insomnia, dependency (IADL) and falls. The latter two could be indicative of frailty, a common syndrome in older people and associated with many of the health variables which have not been entered in multivariate regression analyses. Despite the inability to determine causality and the limitation that most of the assessments were self-reported, strong associations were found in this study. Predictors investigated in this study may help prevent and improve depression in Indonesian older adults, especially those living in Java. Improvement in healthcare, especially in the prevention and rehabilitation of stroke, arthritis, and hearing impairment, concurrent with early detection of depression in these chronic conditions, may help create a better quality of life among Indonesian older adults.

## Figures and Tables

**Table 1:** Classification of socioeconomic and health variables.

**Variable**	**Assessment Question**	**Classification Group**
Sociodemographic	Age in years	(1) 60-69 years (2) 70-79 years (3) ≥80 years
	Sex	(1) Male (2) Female
	Marital status	(1) Married or coinhabiting (2) Never married, separated, widowed, or divorced
	Education status	(1) High (higher than junior high school) (2) Low
	Residential status	(1) Urban (2) Rural
	Living region	(1) Sumatera (2) Java (3) Other regions
Subjective economic status	Please, imagine a six-step ladder where on the bottom stand the poorest people and on the highest step stand the richest people. On which economic step are you today?”	(1) Rich (Step 5 and 6) (2) Medium (Step 3 and 4) (3) Low (Step 1 and 2)
Depression	Depression was assessed using the Centers for Epidemiologic Studies Depression Scale (CES-D 10) question items [27].	(1) Depression (score higher than 10) (2) Normal
Functional ability	Six items of Katz Activity Daily Living (ADL), included bathing, dressing, toileting, transference, continence, and feeding [28]; and Six items of the Lawton Instrumental Activity of Daily Living (IADL), included shopping, food preparation, housekeeping, laundry, taking medications, and handling finance [29].	(1) Independent (2) Dependent
Life satisfaction	Please, think about your life as a whole. How satisfied are you with it?	(1) High (2) Low
Subjective health status	In general, how is your health?	(1) Healthy (2) Unhealthy
Social capital	Presence of activities engaged in the past 12 months, including participation in a community meeting, doing voluntary labor, attending a neighborhood program, and participating in religious activities	(1) High (having at least one of the activities) (2) Low
Tobacco use	Have you ever chewed tobacco, smoked a pipe, smoked self-enrolled cigarettes, or smoked cigarettes/cigars? Do you still have the habit or have you quit?	(1) Never or former (2) Current smoker
Body mass index (BMI)	The body mass index was calculated using height and body weight taken using standard measures and classified using the Asia Pacific classification [30].	(1) Underweight (2) Normal (3) Overweight/obese
Physical activity	Assessed using the International Physical Activity Questionnaire (IPAQ) short version (IPAQ-S7S) [31].	(1) High (2) Moderate (3) Low
Cognitive function	Assessed with items from Telephone Survey Cognitive Status (TICS) [32]. The items include awareness of date and day of the week, self-reported memory question, subtraction of 7s from 100, immediate and delayed word recall of 10 nouns with a total score of 34.	(1) Good cognitive function (2) Possible dementia (score below 8)
Chronic condition	Has a doctor/paramedic/ nurse/midwife ever told you that you had certain disease? These include hypertension, diabetes, stroke, heart disease, arthritis, hypercholesterolemia, cancer, and visual and hearing impairment.	(1) No (2) Yes
Insomnia	Assessed using Patient-Reported Outcomes Measurement Information System (PROMIS) sleep disturbance measure and sleep impairment measure [33].	(1) Normal (2) Insomnia (score higher than 20)
Falls	In the last two years, have you ever fallen?	(1) No (2) Yes

**Table 2:** Variable characteristics.

Variables	Categories	Frequency (n)	Percentage (%)
Total sample		4236	100.0
Depression	No	3545	83.7
	Yes	691	16.3
Age	60-69	2976	70.3
	70-79	1074	25.3
	>= 80	186	4.4
Sex	Male	2105	49.7
	Female	2131	50.3
Marital status	Married/ Coinhabiting	2820	66.6
	Never married, separated, divorced, widowed	1416	33.4
Education	High	634	15.0
	Low	3602	85.0
Subjective economic status	Rich	1120	26.4
	Medium	1690	39.9
	Poor	1426	33.7
Residential status	Urban	2153	50.8
	Rural	2083	49.2
Region	Sumatera	535	12.6
	Jawa	3314	78.2
	Others	386	9.1
Life satisfaction	Yes	3481	82.2
	No	755	17.8
Subjective health status	Healthy	2770	65.4
	Unhealthy	1467	34.6
Social capital	Yes	3488	82.3
	No	748	17.7
Tobacco use	Never, Former	2816	66.5
	Yes	1420	33.5
Physical activities	High	950	22.4
	Moderate	1286	30.4
	Low	2000	47.2
BMI	Underweight	1100	26.0
	Normal	1739	41.0
	Overweight/ Obese	1397	33.0
Cognitive function	Good	3642	86.0
	Possible Dementia	594	14.0
Stroke	No	4133	97.6
	Yes	103	2.4
Heart diseases	No	4061	95.9
	Yes	175	4.1
Hypercholesterolemia	No	3945	93.1
	Yes	291	6.9
Hypertension	No	3067	72.4
	Yes	1169	27.6
Diabetes	No	3952	93.3
	Yes	284	6.7
Arthritis	No	3722	87.9
	Yes	514	12.1
Visual impairment	No	3780	89.2
	Yes	456	10.8
Hearing impairment	No	4150	98.0
	Yes	86	2.0
Cancer	No	4209	99.4
	Yes	27	0.6
Chronic conditions	No	2261	53.4
	One or more	1975	46.6
Insomnia	No	3793	89.5
	Yes	443	10.5
ADL	Independent	3696	87.3
	Dependent	540	12.7
IADL	Independent	3153	74.4
	Dependent	1083	25.6
Falls	No	3728	88-0
	Yes	508	12.0

**Table 3:** Multiple logistic regression analysis of depression among older adults in Indonesia.

Variables	Categories	Depression		Unadjusted Odd Ratio	p-value	Adjusted Odd Ratio	p-value
		No	Yes				
Age	60-69	2490 (70.2)	486 (70.3)	Reference	-	-	-
	70-79	892 (25.2)	182 (26.3)	1.04 (0.87-1.26)	0.661	-	-
	>= 80	163 (4.6)	23 (3.3)	0.73 (0.47-1.14)	0.161	-	-
Sex	Male	1789 (50.5)	316 (45.7)	Reference	-	-	-
	Female	1756 (49.5)	375 (54.3)	1.21 (1.03-1.43)	0.022	-	-
Marital status	Married/ Coinhabiting	2383 (67.2)	437 (63.2)	Reference	-	-	-
	Never married, separated, divorced, widowed	1162 (32.8)	255 (36.8)	1.2 (1.01-1.42)	0.039	-	-
Education	High	564 (15.9)	70 (10.1)	Reference	-	-	-
	Low	2981 (84.1)	621 (89.9)	1.68 (1.29-2.19)	<0.001	1.12 (0.84-1.49)	0.45
Subjective economic status	Rich	997 (28.1)	123 (17.8)	Reference	-	-	-
	Medium	1437 (40.5)	252 (36.5)	1.42 (1.13-1.79)	0.003	1.34 (1.05-1.76)	0.018
	Poor	1110 (31.3)	316 (45.7)	2.3 (1.84-2.89)	<0.001	1.64 (1.28-2.11)	<0.001
Residential status	Urban	1791 (50.5)	362 (52.3)	Reference	-	-	-
	Rural	1753 (49.5)	330 (47.7)	0.93 (0.79-1.1)	0.389	-	-
Region	Sumatera	474 (13.4)	62 (9)	Reference	-	-	-
	Java	2771 (78.1)	544 (78.6)	1.51 (1.14-2)	0.004	1.8 (1.33-2.44)	<0.001
	Others	301 (8.5)	86 (12.4)	2.2 (1.54-3.14)	<0.001	2.25 (1.52-3.31)	<0.001
Life satisfaction	Yes	3007 (84.8)	475 (68.6)	Reference	-	-	-
	No	538 (15.2)	217 (31.4)	2.55 (2.12-3.07)	<0.001	1.75 (1.42-2.17)	<0.001
Subjective health status	Healthy	2455 (69.3)	314 (45.4)	Reference	-	-	-
	Unhealthy	1090 (30.7)	377 (54.6)	2.07 (2.29-3.19)	<0.001	1.85 (1.53-2.24)	<0.001
Social capital	Yes	2925 (82.5)	563 (81.4)	Reference	-	-	-
	No	620 (17.5)	129 (18.6)	1.08 (0.88-1.33)	0.471	-	-
Tobacco use	Never, Former	2374 (67)	442 (63.9)	Reference	-	-	-
	Yes	1171 (33)	250 (36.1)	1.15 (0.99-1.36)	0.114	-	-
Physical activities	High	780 (22)	169 (24.5)	Reference	-	-	-
	Moderate	1080 (30.5)	206 (29.8)	0.88 (0.7-1.1)	0.251	-	-
	Low	1685 (47.5)	316 (45.7)	0.86 (0.7-1.06)	0.159	-	-
BMI	Underweight	897 (25.3)	204 (29.5)	1.24 (1.01-1.51)	0.038	-	-
	Normal	1469 (41.4)	270 (39.1)	Reference	-	-	-
	Overweight/ Obese	1179 (33.3)	217 (31.4)	1 (0.83-1.22)	0.976	-	-
Cognitive function	Good	3063 (86.4)	579 (83.8)	Reference	-	-	-
	Possible Dementia	482 (13.6)	112 (16.2)	1.23 (0.99-1.54)	0.067	-	-
Stroke	No	3477 (98.1)	656 (94.9)	Reference	-	-	-
	Yes	68 (1.9)	35 (5.1)	2.75 (1.81-4.16)	<0.001	2.45 (1.6-3.74)	<0.001
Heart diseases	No	3409 (96.2)	652 (94.4)	Reference	-	-	-
	Yes	136 (3.8)	39 (5.6)	1.48 (1.03-2.14)	0.035	-	-
Hypercholesterolemia	No	3302 (93.1)	643 (93.1)	Reference	-	-	-
	Yes	243 (6.9)	48 (6.9)	1.02 (0.74-1.4)	0.925	-	-
Hypertension	No	260 (73.3)	467 (67.6)	Reference	-	-	-
	Yes	945 (26.7)	224 (32.4)	1.32 (1.12-1.57)	0.002	1.14 (0.95-1.37)	0.155
Diabetes	No	3301 (93.1)	651 (94.2)	Reference	-	-	-
	Yes	244 (6.9)	40 (5.8)	0.84 (0.59-1.18)	0.31	-	-
Arthritis	No	3154 (89)	568 (82.2)	Reference	-	-	-
	Yes	390 (11)	123 (17.8)	1.76 (1.41-2.19)	<0.001	1.59 (1.27-2)	<0.001
Visual impairment	No	3187 (89.9)	592 (85.7)	Reference	-	-	-
	Yes	357 (10.1)	99 (14.3)	1.49 (1.17-1.89)	0.001	1.24 (0.96-1.59)	0.096
Hearing impairment	No	3484 (98.3)	666 (96.4)	Reference	-	-	-
	Yes	61 (1.7)	25 (3.6)	2.12 (1.32-3.4)	0.002	1.69 (1.03-2.77)	0.036
Cancer	No	3519 (99.3)	690 (99.9)	Reference	-	-	-
	Yes	26 (0.7)	1 (0.1)	0.16 (0.02-1.46)	0.104	-	-
Chronic conditions	No	1941 (54.8)	320 (46.3)	Reference	-	-	-
	One or more	1604 (45.2)	371 (53.7)	1.4 (1.19-1.65)	<0.001	1.15 (0.96-1.39)	0.14
Insomnia	No	3324 (93.8)	469 (67.9)	Reference	-	-	-
	Yes	221 (6.2)	222 (32.1)	7.13 (5.78-8.79)	<0.001	5.3 (4.24-6.62)	<0.001
ADL	Independent	3153 (88.9)	543 (78.6)	Reference	-	-	-
	Dependent	392 (11.1)	148 (21.4)	2.2 (1.78-2.71)	<0.001	1.25 (0.97-1.61)	0.081
IADL	Independent	2735 (77.2)	418 (60.5)	Reference	-	-	-
	Dependent	810 (22.8)	273 (39.5)	2.21 (1.86-2.62)	<0.001	1.68 (1.37-2.05)	<0.001
Falls	No	384 (10.8)	124 (17.9)	Reference	-	-	-
	Yes	3161 (89.2)	567 (82.1)	1.81 (1.45-2.26)	<0.001	1.57 (1.23-2.01)	<0.001

## Data Availability

Data employed in this study are publicly available by registering requests at RAND (https://www.rand.org/ well-being/social-and-behavioral-policy/data/FLS/IFLS.html).

## LIST OF ABBREVIATIONS

ADL: = Activity Daily Living
AOR: = Adjusted Odds Ratio
CES-D 10: = Center for Epidemiologic Studies Depression Scale
EAs: = Enumeration Areas
GDS: = Geriatric Depression Scale
IADL: = Instrumental Activity in Daily Living
IFLS-5: = Indonesia Family Life Survey
MMWR: = Mortality and Morbidity Weekly Report
ADL: = Activity of Daily Living
IADL: = Instrumental Activity of Daily Living
IPAQ: = International Physical Activity Questionnaire
TICS: = Telephone Survey of Cognitive Status
